# Melioidosis in the remote Katherine region of northern Australia

**DOI:** 10.1371/journal.pntd.0010486

**Published:** 2022-06-13

**Authors:** Kay Hodgetts, Mariana Kleinecke, Celeste Woerle, Mirjam Kaestli, Richard Budd, Jessica R. Webb, Linda Ward, Mark Mayo, Bart J. Currie, Ella M. Meumann

**Affiliations:** 1 Department of Infectious Diseases, Wellington Regional Hospital, Wellington, New Zealand; 2 Department of Infectious Diseases, Royal Darwin Hospital, Darwin, Northern Territory, Australia; 3 Global and Tropical Health Division, Menzies School of Health Research, Charles Darwin University, Darwin, Northern Territory, Australia; 4 Katherine District Hospital, Katherine, Northern Territory, Australia; 5 Department of Microbiology & Immunology, The University of Melbourne at The Peter Doherty Institute for Infection and Immunity, Melbourne, Victoria, Australia; University of Florida, UNITED STATES

## Abstract

Melioidosis is endemic in the remote Katherine region of northern Australia. In a population with high rates of chronic disease, social inequities, and extreme remoteness, the impact of melioidosis is exacerbated by severe weather events and disproportionately affects First Nations Australians. All culture-confirmed melioidosis cases in the Katherine region of the Australian Top End between 1989–2021 were included in the study, and the clinical features and epidemiology were described. The diversity of *Burkholderia pseudomallei* strains in the region was investigated using genomic sequencing. From 1989–2021 there were 128 patients with melioidosis in the Katherine region. 96/128 (75%) patients were First Nations Australians, 72/128 (56%) were from a very remote region, 68/128 (53%) had diabetes, 57/128 (44%) had a history of hazardous alcohol consumption, and 11/128 (9%) died from melioidosis. There were 9 melioidosis cases attributable to the flooding of the Katherine River in January 1998; 7/9 flood-associated cases had cutaneous melioidosis, five of whom recalled an inoculating event injury sustained wading through flood waters or cleaning up after the flood. The 126 first-episode clinical *B*. *pseudomallei* isolates that underwent genomic sequencing belonged to 107 different sequence types and were highly diverse, reflecting the vast geographic area of the study region. In conclusion, melioidosis in the Katherine region disproportionately affects First Nations Australians with risk factors and is exacerbated by severe weather events. Diabetes management, public health intervention for hazardous alcohol consumption, provision of housing to address homelessness, and patient education on melioidosis prevention in First Nations languages should be prioritised.

## Author summary

Melioidosis, caused by the environmental bacterium *Burkholderia pseudomallei*, disproportionately affects Australian First Nations peoples in the Northern Territory of Australia. The Katherine region has some of the highest rates of homelessness in Australia, and social inequity impacts health outcomes for First Nations people whose access to care is further complicated by remoteness. In this study, we describe the clinical features and epidemiology of melioidosis in the Katherine region over a 32-year period. Almost three quarters of melioidosis cases were First Nations Australians, over half lived in a very remote region, and diabetes and hazardous alcohol consumption were common risk factors. Following a severe flooding event in the region in 1998, a spike in cases of melioidosis was seen, the majority presenting as skin and soft tissue infections. The *B*. *pseudomallei* isolates in the study were extremely genetically diverse, reflecting the large geographic area of the Katherine region. With predicted climate change-driven increases in severe weather events and subsequent increases in melioidosis cases, public health priorities in the region should include addressing high rates of homelessness and hazardous alcohol consumption, optimisation of diabetes management, and ongoing education in First Nations languages regarding prevention of *B*. *pseudomallei* exposure.

## Introduction

Melioidosis is the disease caused by infection with the environmental bacterium, *Burkholderia pseudomallei*, which is found in soil and surface water predominantly in tropical regions. Melioidosis ranges in severity from asymptomatic infection to septic shock, and it remains an important cause of fatal pneumonia and sepsis in northern Australia [1]. Most melioidosis cases occur during the wet season, with clinical presentation often preceded by identifiable occupational or recreational exposures; percutaneous inoculation and inhalation are thought to be the most common modes of acquisition [2]. Important risk factors for melioidosis include diabetes and hazardous alcohol consumption [1].

The Katherine region is part of the ‘Top End’ of the Northern Territory, Australia, and is 320 km south of the northern coastline. The region is sparsely populated, spanning 340,000 km^2^ with a population of ~18,600 people, and the entire region is classified as either ‘remote’ or ‘very remote’ [3]. ~6,300 people live in the town of Katherine and the remainder live in remote First Nations communities. Just over half of the region’s population are Australian First Nations people belonging to over twenty tribal nations. First Nations people are affected by social and health inequities, and the homelessness rate in Katherine is 31 times the national average [4–6]. There are high rates of chronic disease, the management of which is further complicated by remoteness, and age-adjusted mortality is the highest in Australia [4, 7]. The link between socioeconomic disadvantage and melioidosis has recently been emphasised in a study from far north Queensland, Australia [8].

The Top End of the Northern Territory including the Katherine region is part of the wet-dry tropics and experiences a monsoonal wet season from November to April each year, and cyclones during this period are common. Tropical Cyclone Les occurred in January 1998; there was 380 mm rainfall in a 48-hour period resulting in two metres of flood water inundating the town of Katherine on 27 January 1998 [9]. This event was followed by a spike in melioidosis cases.

Here we describe the risk factors, clinical presentations, and outcomes of patients with melioidosis in the Katherine region from 1989–2021, including the spike in cases associated with the flooding event, and we describe the genomic diversity of their *B*. *pseudomallei* isolates.

## Methods

### Ethics statement

The study was approved by the Human Research Ethics Committee of the Northern Territory Department of Health and Menzies School of Health Research (approval number 02/38). Consent was not obtained from study participants as inclusion did not impact clinical care.

### Melioidosis cases and definitions

**Fig 1:**
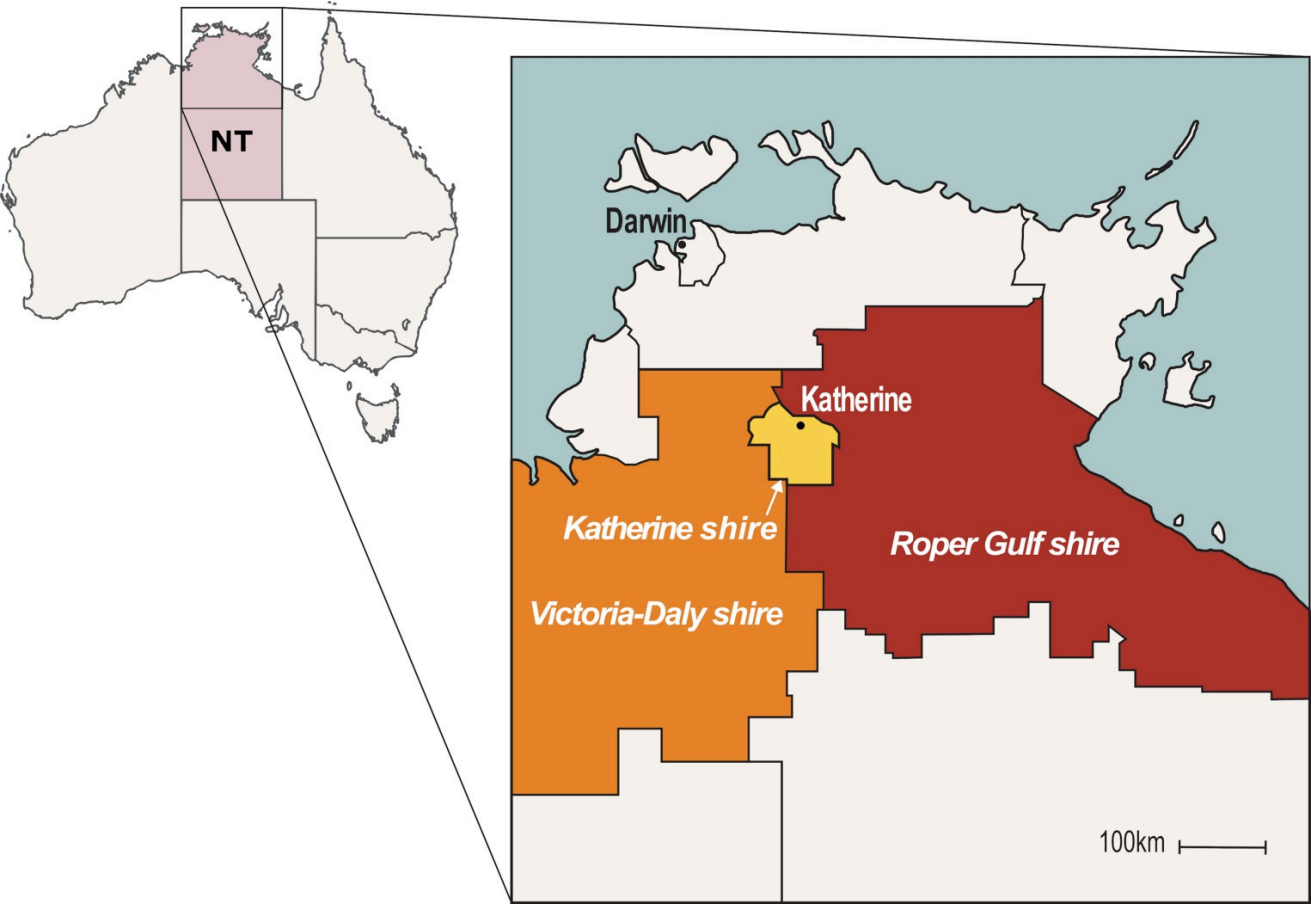
Map of the Katherine region of northern Australia. **NT = Northern Territory. Map adapted from** https://www.lgant.asn.au/councils-2/.

The Darwin Prospective Melioidosis Study (DPMS) has documented all culture-confirmed cases of melioidosis in the Top End of the Northern Territory of Australia since 1989 [1]. In this study we included all DPMS cases from the Katherine region, that is, a subset of the DPMS. The Katherine region includes the town of Katherine (Katherine shire), and extends to the Gulf of Carpentaria to the east (Roper Gulf shire), and to the border of Western Australia to the west (Victoria-Daly shire; Fig 1). In this manuscript, the terms ‘First Nations’ or ‘Indigenous’ are respectfully used to refer to Australian Aboriginal and Torres Strait Islander peoples.

We defined the annual number of cases as the number of cases from 1 October until 30 September, encompassing the wet season from November-April. Acute presentations were defined as having had symptoms for <2 months and chronic infections for ≥2 months prior to presentation. Recurrent melioidosis was defined as a new culture-confirmed episode of melioidosis after treatment completion. Definitions for melioidosis risk factors and septic shock were as previously defined [1].

All cases were managed by or in discussion with the Infectious Diseases team at Royal Darwin Hospital located in the Northern Territory capital, 320 km to the north of Katherine, and were treated in accordance with local antibiotic guidelines which comprise an initial period of intravenous treatment with ceftazidime or meropenem, followed by at least three months of oral eradication therapy with either trimethoprim-sulfamethoxazole or doxycycline [10].

### Environmental samples

Seventeen *B*. *pseudomallei* isolates from the Katherine River were included in the study. Water samples were collected from the river between 2012–2017 in 200 mL bottles and *B*. *pseudomallei* was cultured from these as previously described [11].

### Statistical analysis

Analysis of clinical data was done using R v3.5.3. Two-tailed Fisher’s exact test was used to compare categorical variables and considered significant if *P*<0.05. Population estimates for the Katherine region were obtained from the Australian Bureau of Statistics (https://stat.data.abs.gov.au/), and rainfall readings for Katherine town from the Bureau of Meteorology (http://www.bom.gov.au/).

### Bioinformatics analysis

We sequenced clinical *B*. *pseudomallei* isolates including one isolate per melioidosis episode, and the seventeen water *B*. *pseudomallei* isolates. Accessions for *B*. *pseudomallei* genomes included in this study are listed in S1 Table. We undertook whole genome sequencing using the Illumina platform, and conducted *in silico* multilocus sequence typing (MLST; https://pubmlst.org/bpseudomallei/). Multiple sequence alignment and variant calling was done with Snippy v4.3.6 (https://github.com/tseemann/snippy) with thresholds of ≥10× coverage and ≥90% variant prevalence. MSHR1153 (GenBank accessions CP009271.1 and CP009272.1) was used as the reference genome. Phylogenetic analysis was done with IQ-TREE v2.0 using a generalized time-reversible model, four gamma categories, 1,000 ultrafast bootstrap replicates and 1,000 approximate likelihood-ratio test replicates [12]. The tree was visualized and annotated with the ggtree package in R v3.5.3 [13].

## Results

### Melioidosis cases in the Katherine region

**Fig 2:**
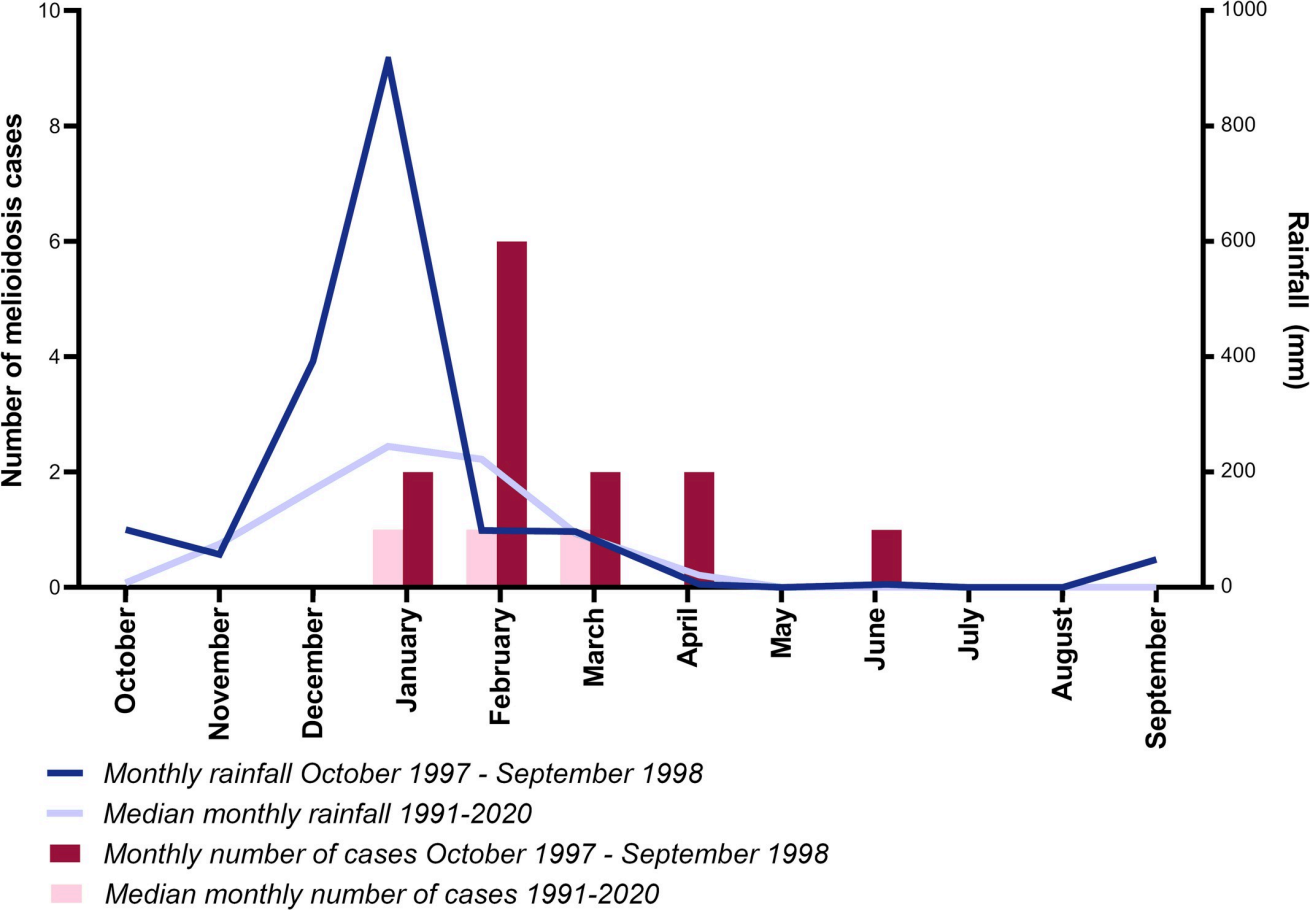
Monthly total rainfall and melioidosis cases in Katherine 1997–1998, and median total rainfall and melioidosis cases in Katherine 1991–2020.

There were 128 cases of melioidosis in the Katherine region from 1989–2021. The median number of annual cases was 3.5 (range 0–13 cases), with the peak of 13 cases occurring between October 1997 and September 1998, associated with the flooding of the Katherine River (Fig 2). The estimated incidence of melioidosis in the Katherine region in 1997–1998 was 69 per 100,000 population based on population size of ~18,600 people, compared to the median annual incidence of 20 per 100,000 from 2001–2021.

The median age of melioidosis cases in the Katherine region was 45.5 years (range 1–74 years) with just 10 (7.8%) children aged ≤ 15 years. 96/128 (75%) were First Nations people compared to 663/1218 (52.6%) in the DPMS overall (Table 1). The majority of melioidosis cases in the very remote Roper Gulf and Victoria-Daly shires were First Nations (62/72 [86.1%]) however First Nations people also accounted for more than half of cases in Katherine town shire (34/56 [60.7%]). There was no difference in the proportion of Roper Gulf/Victoria-Daly versus Katherine shire cases with diabetes (39/72 [54.2%]) versus 29/56 [51.8%]) or hazardous alcohol consumption (33/72 [45.8%]) versus 24/56 [42.9%]).

**Table 1:** Melioidosis cases in the Katherine region.

	Katherine region and overall Top End		Katherine region flood- and non-flood-associated cases	
	Katherine region, number (%)	Top End, number (%)	Flood-associated, number (%)	Non-flood-associated, number (%)
**Demographics**				
Australian First Nations	96/128 (75%)	663/1218 (54.4%)	2/9 (22.2%)	94/119 (79.0%)
Median age in years (range)	45.5 (1–74)	50 (0–97)	39 (22–66)	46 (1–74)
Male sex	71/128 (55.5%)	762/1218 (62.6%)	5/9 (55.6%)	66/119 (55.5%)
**Risk factors**				
Diabetes	68/128 (53.1%)	663/1218 (54.4%)	2/9 (22.2%)	66/119 (55.5%)
Hazardous alcohol consumption	57/128 (44.5%)	732/1218 (60.1%)	1/9 (11.1%)	56/119 (47.1%)
**Primary clinical presentation**				
Pneumonia	50/128 (39.1%)	633/1218 (51.9%)	2/9 (22.2%)	48/119 (40.3%)
Skin and/or soft tissue infection	35/128 (27.3%)	208/1218 (17.1%)	7/9 (77.8%)	28/119 (23.5%)
Bacteremia with no focus	17/128 (13.3%)	136/1218 (11.2%)	0	17/119 (14.3%)
Genitourinary tract infection	10/128 (7.8%)	145/1218 (11.9%)	0	10/119 (8.4%)
Bone and/or joint infection	8/128 (6.2%)	46/1218 (3.8%)	0	8/119 (6.7%)
Central nervous system infection	6/128 (4.7%)	21/1218 (1.7%)	0	6/119 (5.0%)
Other	2/128* (1.6%)	29/1218 (2.4%)	0	2/119 (1.7%)
**Severity**				
Bacteremia	64/127 (50.4%)	671/1205 (55.7%)	2/9 (22.2%)	62/118 (52.5%)
Intensive care unit admission	38/128 (29.7%)	289/1218 (23.7%)	1/9 (11.1%)	37/119 (31.1%)
Septic shock	34/128 (26.6%)	248/1218 (20.4%)	1/9 (11.1%)	33/119 (27.7%)
Death due to melioidosis	11/128 (8.6%)	136/1218 (11.2%)	1/9 (11.1%)	10/119 (8.4%)

*Liver abscess

**Fig 3:**
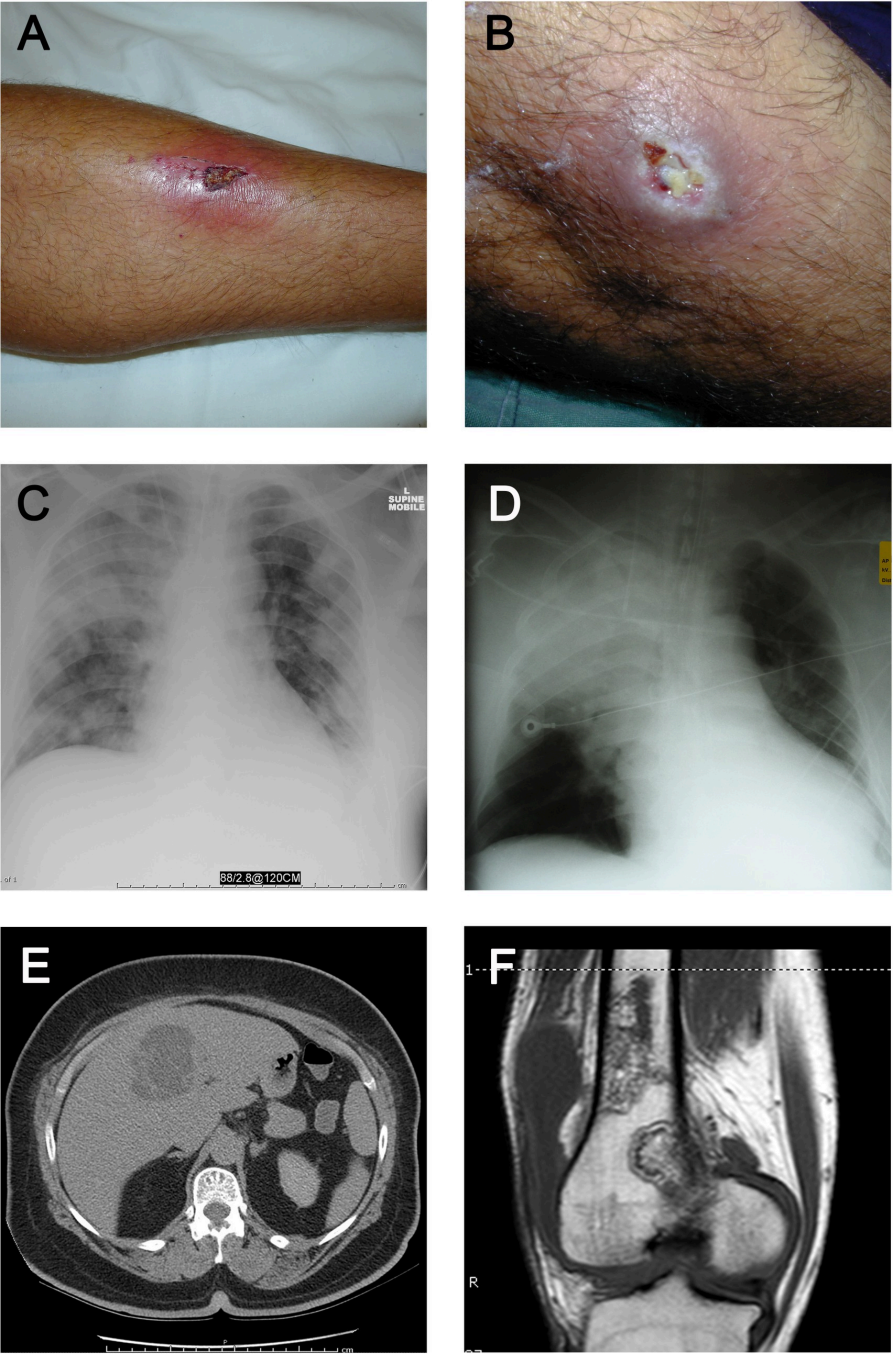
Clinical and radiologic images of melioidosis cases in the Katherine region. (A) Cutaneous melioidosis; (B) Cutaneous melioidosis; (C) Non-fatal bacteremic melioidosis pneumonia; (D) Fatal bacteremic melioidosis pneumonia; (E) Computed tomography scan demonstrating a liver abscess; (F) Magnetic resonance imaging scan demonstrating osteomyelitis of the femur. Photos taken by Prof Bart Currie.

As noted in the larger DPMS study [1], pneumonia was the most common primary presentation (50/128 [39.1%]) (Table 1 and Fig 3) Skin and/or soft tissue infection accounted for a larger proportion of cases (35/128 [27.3%]) than in the DPMS overall (208/1218 [17.1%]; Table 1 and Fig 3). Prostatic abscesses were present in 12/71 (16.9%) men, either as the primary presentation (7 cases) or secondary to another primary presentation (5 cases). *B*. *pseudomallei* was isolated from blood cultures in 64/127 (50.4%), 34/128 (26.6%) patients developed septic shock, 38/128 (29.7%) required intensive care support, and 11/128 (8.6%) died from melioidosis. Septic shock was more common among very remote Roper Gulf/Victoria-Daly shire patients (26/72 [36.1%]) than Katherine town shire patients (8/56 [14.3%], *P* = 0.008), however mortality was similar in the two populations (6/72 [8.3%] versus 5/56 [8.9%]).

Seven patients had recurrent episodes of culture-confirmed melioidosis after treatment completion; five cases relapsed, one patient with underlying chronic lymphocytic leukaemia had a new infection six years after initial presentation, and one patient with hazardous alcohol consumption had a new infection eighteen years after initial presentation. The median time to relapse from initial presentation was 13 months (range 7–22 months), and all five patients with relapsed melioidosis did not complete oral eradication therapy. All except for one patient who relapsed lived in a remote community.

### Melioidosis cases associated with the Katherine floods

There were nine cases of melioidosis diagnosed between 2 February and 7 April 1998 associated with the Katherine floods (Tables 1 and 2). Cutaneous melioidosis was the most common presentation, seen in 7/9 cases, and 6/9 cases had no identifiable risk factor for melioidosis. Five cases with cutaneous melioidosis were able to recall an inoculating event; these were injuries sustained wading through flood waters or cleaning up after the floods. Three cases of cutaneous melioidosis were diagnosed >1 month after the flooding event but provided a clear history of a flood-related injury; each of these cases had received prior treatment for presumptive Gram-positive organisms prior to isolation of *B*. *pseudomallei*.

**Table 2:** Katherine flood-associated melioidosis cases.

Demographics*	Risk factors	Inoculating event	Incubation period	Clinical presentation	Outcome
65F, non-Indigenous	Diabetes, chronic lung disease	Unknown	3 days	Acute pneumonia with septic shock	Died
53M, First Nations	Diabetes	Walked 3km through flood waters to recover belongings	1 day	Acute bacteraemic pneumonia	Survived
22F, non-Indigenous	None	Cut hand on glass cleaning up after floods	1 day	Finger abscess requiring incision and drainage	Survived
65F, First Nations	None	Unknown	Unknown	Lower leg ulcer	Survived
39F, non-Indigenous	None	Unknown	Unknown	Pustule overlying knee	Survived
24M, non-Indigenous	None	Cut lower leg in flood waters	10 days	Infected leg wound	Survived
53M, non-Indigenous	None	Cut shin on caravan tow bar in flood waters	Unknown	Chronic ulcer on shin	Survived
38M, non-Indigenous	None	Cut finger on barbed wire and fell into flood waters	Unknown	Chronic wound on finger	Survived
37M, non-Indigenous	Hazardous alcohol consumption	Cut lower leg on ceramic pot in flood waters	5 days	Lower leg ulcer	Survived

*Age in years, sex (M = male, F = female), ethnicity

### Multilocus sequence typing of B. pseudomallei isolates from the Katherine region

From the 128 melioidosis cases, 133 *B*. *pseudomallei* isolates were sequenced including seven isolates from recurrent infections. Seventeen water isolates from the Katherine River were also included. The 55 first-episode clinical isolates from Katherine town shire belonged to 46 different sequence types (STs), with the most common being ST734 (5 cases). Nine STs found in the Katherine River were also isolated from clinical samples; these included ST109, ST150, ST266, ST294, ST722, ST734, ST738, ST746, and ST1594. The nine *B*. *pseudomallei* isolates from the flood-associated cases belonged to nine different STs, with two of these STs (ST294 and ST722) also being found in the Katherine River. There were 66 different STs among the 71 first-episode clinical isolates from the very remote Roper Gulf and Victoria-Daly shires. Eighteen of the STs found in the Katherine region have previously been found in melioidosis patients in other Top End regions. This includes ST109; Katherine ST109 isolates have previously been found to be unrelated to Darwin ST109 isolates at the whole genome level [14].

### Phylogenetic analysis of B. pseudomallei isolates from the Katherine region

**Fig 4:**
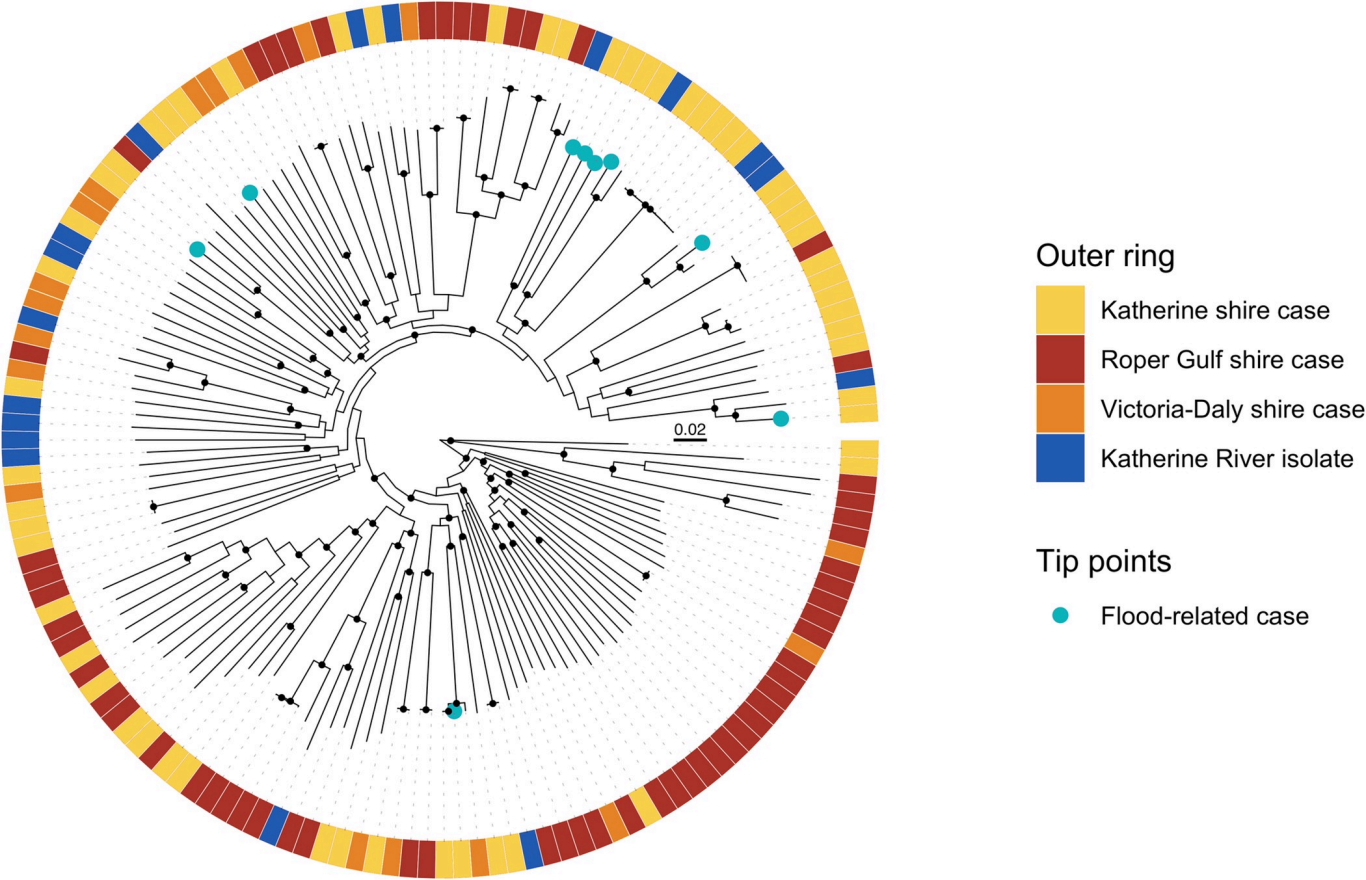
Midpoint-rooted maximum-likelihood phylogeny of clinical and environmental *B*. *pseudomallei* genomes from the Katherine region. Nodes with approximate likelihood ratio >95 and ultrafast bootstrap >95 are marked with a black circle. Scale bar indicates substitutions/site.

A maximum-likelihood phylogenetic tree demonstrated that the *B*. *pseudomallei* isolates causing melioidosis in the Katherine region are very diverse, with a median pairwise SNP distance of 20,092 SNPs (Fig 4). The *B*. *pseudomallei* genomes from clinical cases in different geographical areas within the Katherine region were phylogenetically interspersed. The ST109 river isolate was separated from a clinical ST109 isolate by 56 SNPs, and the ST734 river isolate was separated from ST734 clinical isolates by 84–112 SNPs. River isolates belonging to STs 150, 266, 294, 722, and 738 were located on the same clades as clinical isolates belonging to the same STs, but were distantly related and separated by 5,516–9,437 SNPs. Consistent with the MLST findings, genomes from flood-associated cases were diverse and located on distantly related clades in the phylogeny.

## Discussion

Here, we have described the epidemiology and clinical features of melioidosis in the Katherine region, a remote, sparsely populated region of the Australian Top End. The annual incidence of melioidosis on average was 20 per 100,000 in the Katherine region, similar to the Top End overall [1]. Homelessness in the town of Katherine and the outdoor lifestyle associated with remote living in the region are likely to lead to exposure to *B*. *pseudomallei* [6]. Homelessness was previously hypothesised to contribute to increased melioidosis incidence in urban Darwin following the Northern Territory Emergency Response (‘The Intervention’) initiated by the Australian Government in 2007, which was associated with movement from remote to urban settings [15]. The prevalence of known melioidosis risk factors is high in the Katherine region; diabetes prevalence is >8%, and like other Northern Territory regions the prevalence of hazardous alcohol consumption is well above the national average [16, 17].

Public health messaging for melioidosis prevention in those with risk factors includes advice to wear shoes outdoors to prevent inoculating injuries, and to stay indoors during storms to avoid inhalation of *B*. *pseudomallei*. These messages have been translated into Katherine region First Nations languages including Eastside Kriol and Warlpiri, and are played on local radio each wet season [18]. Further evaluation of the effectiveness of public health messaging is needed, as it is very likely that barriers exist to enacting these preventive measures.

In Thailand, qualitative research identified multiple barriers to behaviour change to reduce melioidosis risk [19], and a large randomised-controlled trial of a behaviour change support group for melioidosis prevention in people with diabetes did not demonstrate a reduction in *B*. *pseudomallei* infection [20]. In Far North Queensland, Australia, low socioeconomic status has been found to be associated with melioidosis risk factors and *B*. *pseudomallei* infection, and was an independent risk factor for death due to melioidosis [8]. For those at greatest social and health risk in Katherine, community-led case management led to a reduction in hospital admissions and to increased engagement with primary healthcare [6]. Over the long term, a program such as this could lead to a reduction in melioidosis due to its effect on risk factors.

The Katherine region has a 60-bed hospital which services an area of 340,000km^2^ and lacks an intensive care unit; critically ill patients with suspected or confirmed melioidosis are therefore transferred to Royal Darwin Hospital by road or air ambulance. Septic shock was twice as common among melioidosis cases from very remote regions (36%, versus 14% for Katherine town shire) however there was no difference in mortality, and mortality overall was lower (9%) than for the Top End overall for the same time period (11%) [1]. This suggests that for the most part, remote clinics provide early effective treatment for sepsis with intravenous fluid resuscitation and antimicrobial therapy (usually including intravenous ceftriaxone 2g) while awaiting aeromedical retrieval.

On average, the inland Katherine region is drier than coastal Darwin where most Top End melioidosis cases occur (median annual rainfall 964 mm for Katherine versus 1729 mm for Darwin) [1, 21]. Studies in the Northern Territory have shown a clear association between increased rainfall and increased melioidosis cases [15, 22–24]. Consistent with this, the 1998 Katherine flood event was associated with a striking increase in melioidosis cases. More than half of flood-associated cases recalled an inoculating event, which was usually an injury sustained in the flood waters. Most flood-associated cases presented with non-healing ulcers, did not have melioidosis risk factors, and were not systemically unwell–all typical features of cutaneous melioidosis [25]. Although it has previously been hypothesised that severe weather events including cyclones and typhoons can lead to inhalational melioidosis [26, 27], we did not observe evidence of this associated with Tropical Cyclone Les and the flooding of the Katherine River.

The clinical *B*. *pseudomallei* isolates in this study were extremely diverse, consistent with the sparsely populated, large geographic size of the Katherine region. This contrasts with observations in the Northern Territory capital city Darwin, where *B*. *pseudomallei* is diverse but there are five dominant *B*. *pseudomallei* STs [1, 11, 28]. The nine cases associated with the Katherine floods all belonged to different STs, and the *B*. *pseudomallei* isolates from the Katherine River were similarly diverse. During periods of heavy rainfall, *B*. *pseudomallei* proliferates in soil and surface water and is washed into drains, creeks, and rivers [29]. The *B*. *pseudomallei* strains found in a river system may therefore reflect the diversity of isolates in a large catchment area, and rivers may act as conduits for *B*. *pseudomallei* dispersal [11, 30]. Our findings are consistent with a previous study that found that in melioidosis-endemic regions, *B*. *pseudomallei* isolates associated with melioidosis clusters following severe weather events are not clonal [22].

In contrast to the millennia of successful adaptation to changing environments, First Nations Australians’ postcolonial circumstances of low socioeconomic status, household crowding and high rates of comorbidities militate against adaptation to anthropogenic climate change [31, 32]. A predicted increase in the frequency of severe weather events is likely to lead to increased melioidosis cases in the Top End including the Katherine region in the future [23, 33, 34]. Addressing melioidosis risk is therefore key. This includes public health intervention for hazardous alcohol consumption [16], optimisation of glycaemic control in those with diabetes, greater support and commitment for partnerships and innovative programs to address the high rates of homelessness in the region [5, 6], and working with remote clinics to provide education on preventing exposure to *B*. *pseudomallei*.

## Supporting information

S1 Table Sequence Read Archive accessions for *Burkholderia pseudomallei* genomes included in this study. (XLSX)
